# Organoid technology in disease modelling, drug development, personalized treatment and regeneration medicine

**DOI:** 10.1186/s40164-018-0122-9

**Published:** 2018-12-05

**Authors:** Hanxiao Xu, Ying Jiao, Shuang Qin, Weiheng Zhao, Qian Chu, Kongming Wu

**Affiliations:** Department of Oncology, Tongji Hospital of Tongji Medical College, Huazhong University of Science and Technology, 1095 Jiefang Avenue, Wuhan, 430030 China

**Keywords:** Organoids, Development, Infectious diseases, Genetic diseases, Cancer, Drug development, Precision medicine, Regeneration medicine

## Abstract

Organoid technology bridges the gap between conventional two-dimensional cell line culture and in vivo models. The near-physiological technology can virtually recapitulates organ development and human diseases, such as infectious diseases, genetic abnormality and even cancers. In addition, organoids can more accurately predict drug responses, and serve as an excellent platform for drug development, including efficacy evaluation, toxicity testing and pharmacokinetics analysis. Furthermore, organoids can also be exploited to explore the possible optimized treatment strategies for each individual patient. Besides, organoid technology is a promising strategy for regeneration medicine and transplantation use, which can overcome the deficiency in the supply of healthy donor tissues and inherent immunological rejection through establishing isogenic organoids from minuscule amounts of patient biopsies. Collectively, organoids hold enormous potential for clinical applications and bring basic research closer to clinical practice. In this review, we described common organoid lines, summarized the potential clinical applications, and outlined the current limitations.

## Background

Two-dimensional cell line culture and animal models have long been exploited to study embryonic development and human diseases. However, diverse drawbacks make these conventional models to be suboptimal. For instance, cell lines display the inability in modeling immune system, stromal components and organ specific functions as well as the gradual loss in genetic heterogeneity of original cells after many passages [1]. Animal models possess different structures and physiology [2] as well as experience species-specific organ development and pathogenesis [3].

**Fig. 1:**
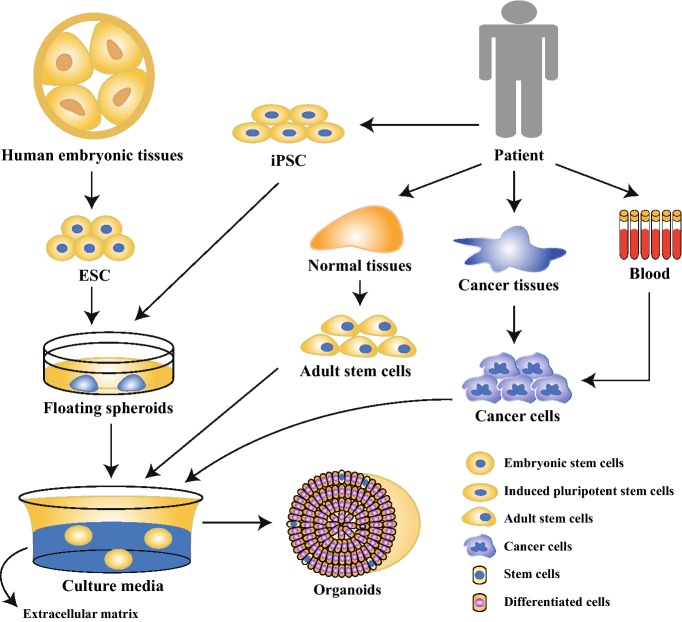
The establishment of organoids. Organoids can be developed from ESCs, iPSCs, adult stem cells and cancer cells in specific 3D culture medium. ESCs embryonic stem cells, iPSCs induced pluripotent stem cells, 3D three-dimensional

During the past decades, organoid technology, an innovative three-dimensional (3D) model, has risen rapidly and become more and more prevalent among researchers. Organoids are 3D tissues in miniature in vitro, which are specific to the parent counterparts in vivo [4]. These amazing 3D constructs, which can be established from embryonic stem cells (ESCs), induced pluripotent stem cells (iPSCs), adult stem cells and even tumor cells in 3D culture system (Fig. 1), contain multifarious cell types of original organs and mimic the derived organs in both architecture and function to a great degree [5].

**Fig. 2:**
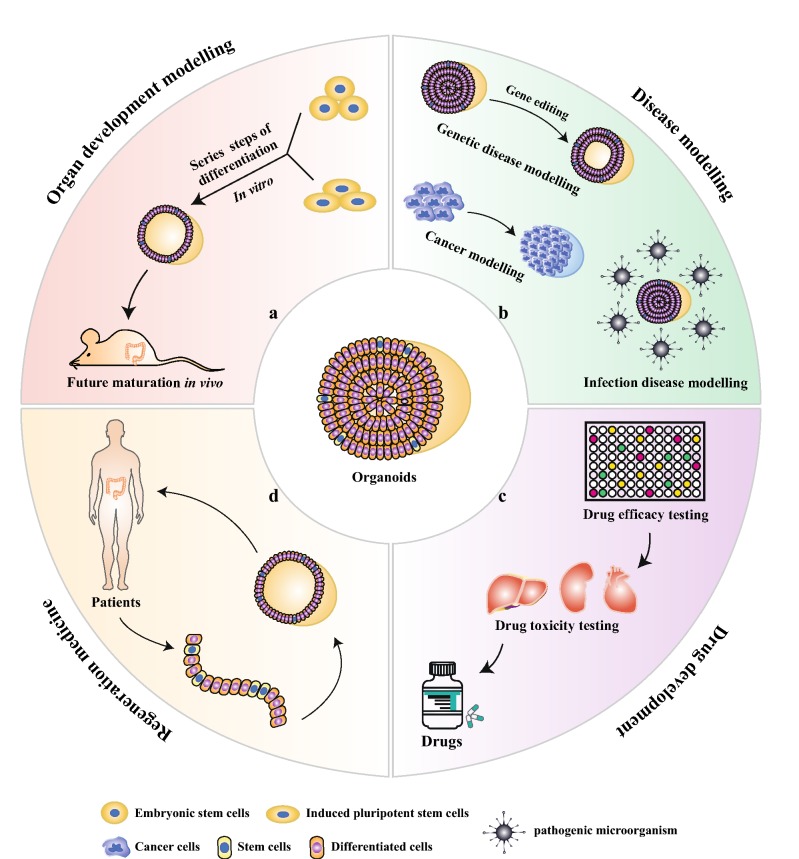
The potential applications of organoids. Organoids can be exploited to model organ development (**a**) and human diseases (**b**), including infectious diseases, genetic diseases and cancers. **c** Organoids can also facilitate drug development through testing drug efficacy and toxicity. **d** Regeneration medicine is another potential application of organoids by replacing non-functional organs with healthy organoids

Up to now, healthy organoids have been developed successfully from various organs, such as lung [6–8], gastric [2], intestinal [9], liver [10], pancreas [10, 11], kidney [12–15], prostate [16] and brain [17, 18]. Apart from healthy organoids, organoid biobanks of multiple human cancers have also been established efficiently, including gastrointestinal cancer [19, 20], liver cancer [21], breast cancer [22] and bladder cancer [23]. Organoid technology demonstrates tremendous potential for fundamental research and clinical applications (Fig. 2). Firstly, organoids can be employed to model and study organ development [24] and human diseases, as exemplified by genetic conditions [25, 26], infectious diseases [27, 28] and tumors [29, 30]. Secondly, organoids can be exploited as an excellent platform to evaluate drug efficacy [31] and toxicity [32], ultimately promoting drug development. Thirdly, precision medicine might be another pivotal branch of organoid technology by accurately predicting drug responses [33] and guiding to make optimized treatment strategies for each patient individual. Finally, organoids show regeneration potential after transplantation to animals [34, 35], signifying that it can serve as an alternative approach to tissue replacement strategy for irreversibly progressively diseased or non-functional organs.

In this review, we describe diverse organoid types and focus on potential applications, including organ development, disease modeling, drug development, precision medicine and regeneration medicine. Finally, we outline the current limitations of this amazing technology.

## Current organoid technology

Multiple types of organoids have been efficiently established in specific 3D culture system, including lung organoids [6], gastric organoids [36, 37], intestinal organoids [9], liver organoids [38], pancreatic organoids [39], prostate organoids [16] and brain organoids [40]. The distinct culture components for diverse organoids were displayed in Table 1.

**Table 1:** Culture systems of multiple organoids

Organoid type	Sources	Culture components	Ref.
Lung	Lung alveolar epithelial cells (together with lung fibroblasts)	Matrigel SAGM medium with all additives except epinephrine (Y27632 was added during the first 2 days. Wnt3A, FGF7, FGF10, XAV939 and CHIR99021 were added after the first 2 days)	[6]
Gastric	hPSCs	Matrigel Advanced Dulbecco’s modified Eagle’s medium (DMEM)/F12 with N2, B27, l-glutamine, HEPES, penicillin/streptomycin and EGF (Retinoic acid and Noggin were added for the first 3 days)	[36]
	Lgr5^+^ stem cells	Matrigel Advanced DMEM/F12 with N2, B27, *N*-acetylcysteine, Gastrin, and EGF, R-spondin-1, Noggin, FGF10 and Wnt3A (Y-27632 was added for the first 2 days, and Exendin 4 was added for the differentiation of enteroendocrine lineage)	[37]
Intestinal	ESCs and iPSCs	Matrigel Advanced DMEM/F12, N2, B27, l-glutamine, HEPES, penicillin–streptomycin, EGF and Noggin	[9]
Liver	iPSCs	Matrigel Hepatocyte culture medium supplemented with dexamethasone, oncostatin M, hepatocyte growth factor and SingleQuots	[38]
Pancreas	Pancreatic progenitor from hPSCs	Matrigel MCDB131, glucose, sodium bicarbonate, FAF- bovine serum albumin, l-glutamine, l-ascorbic acid, FGF2, Y27632, insulin–transferrin–selenium–ethanolamine, nicotinamide, FGF10, glucose, indolactam V and the transforming growth factor β-receptor inhibitor SB431542	[39]
Prostate	Luminal and basal prostatic epithelial lineages	Matrigel Advanced DMEM/F12, penicillin/streptomycin, GlutaMAX and Hepes, B27, *N*-acetylcysteine, EGF, the transforming growth factor-beta inhibitor A-83-01, Noggin, R-spondin-1, dihydrotestosterone, nicotinamide, FGF2, FGF10, SB202190 and prostaglandin E2 (Y-27632 is added when passaging with TrypLE)	[16]
Brain	ESCs	Matrigel Advanced DMEM/F12, FGF2, insulin, Shh signaling inhibitor, bone morphogenetic protein, Y-27632, SB431542, FGF19 and stromal cell-derived factor 1	[40]

### Lung organoids

Lung organoids have been successfully established in multiple studies [6–8, 41, 42]. Following definitive endoderm induction and anteriorization through manipulation of developmental signaling pathways, human pluripotent stem cells (hPSCs) ultimately formed lung bud organoids (LBO) with branching morphogenesis in Branching media and Matrigel [7]. In human lung organoids, it was observed that smooth muscle and myofibroblasts surrounded upper airway-like epithelial with immature ciliated cells and basal cells as well as a distal alveolar-like section [8]. In addition, human alveolar epithelial progenitors together with lung fibroblasts, cultured in SAGM media (all additives without epinephrine) and growth factor-reduced Matrigel (phenol-free), developed into functional alveolar epithelial, containing alveolar type 1 (AT1) and AT2 cells [6]. Besides, through regular modulation of the canonical Wnt signaling, iPSCs can finally develop into functional proximal airway organoids via NKX2-1^+^ progenitors [42]. The airway organoids only contain epithelial section with multiple airway epithelial cell types, including ciliated, goblet and basal cells [42]. In another study, combination culture of human somatic primary bronchial epithelial cells, lung fibroblasts and lung microvascular endothelial cells, also led to the generation of airway organoids in Matrigel and PneumaCult-ALI Maintenance/Complete Base Medium, in which there were invasive multicellular luminal structures [41].

### Gastric organoids

Gastric organoids have been efficiently developed in various researches [2, 36]. McCracken and colleagues unprecedentedly reported the de novo establishment of human gastric organoids from hPSCs [36]. Following definitive endoderm induction and foregut spheroid generation, floating foregut spheroids were cultured in specific 3D culture system [36]. It was demonstrated that combination treatment of Wnt3A, fibroblast growth factor 4 (FGF4) and Noggin (a bone morphogenetic protein inhibitor) directed definitive endoderm to foregut, and retinoic acid promoted the conversion from posterior foregut spheroids to sex determining region Y-box 2 (SOX2)/pancreatic and duodenal homeobox 1 (PDX1)^+^ antrum epithelial [36]. In another study, single adult Lgr5^+^ stem cells isolated from gastric gland units can also develop into gastric organoids [37]. For avoiding anoikis, the Rho kinase inhibitor Y-27632 was added during the first 2 days after seeding [37]. For direction to enteroendocrine lineage, Exendin 4 was supplemented [37]. These long-lived gastric organoids resembled mature pyloric epithelium, in which newly formed pyloric-like structure was a single-layered epithelial containing a central glandular lumen full of apoptotic cells surrounded with gastric gland-domain buds [37]. The organoids still maintained this property even during the ninth month of culture with the expression of gastric epithelial markers including gastric intrinsic factor, pepsinogen C and mucin 6 [37].

### Intestinal organoids

The technology to establish intestinal organoids have been developed [9, 43–47]. IPSCs-derived intestinal organoids contained diverse intestinal cells, including intestinal stem cells, enterocytes, goblet cells, enteroendocrine cells, paneth cells, fibroblasts and smooth muscle cells [47]. In addition, intestinal markers and pharmacokinetic-related genes were detected, and some structural features (microvilli and tight junctions) were observed in organoids [47]. Furthermore, these organoids expressed drug transporters, and showed efflux transport activity and the activation of the drug-metabolizing enzyme cytochrome P450 [47]. Human colon organoids have been successfully developed from hPSCs [9]. Colon organoids contained multiple colon-typical cell population, including goblet cells and enteroendocrine cells [9]. After transplantation into mice, colon organoids experienced morphogenesis and maturation to form tissues, which greatly resembled human colon in molecular, cellular and morphologic properties [9]. Organoids contained more cells with enhanced expression of preproglucagon, peptide YYand colon specific hormone insulin-like 5 [9].

### Liver organoids

Liver organoids have been established successfully in a flurry of studies [10, 21, 38, 48–50]. Broutier’s group summarized a detailed protocol of an in vitro 3D culture system for human liver organoids [10]. When transferred from expansion median to differentiation median, liver organoids were endowed with functional hepatocyte characteristics of albumin production, bile acid production and cytochrome activity as well as enhanced expression of typical hepatocyte markers including albumin, cytochromes and α-1-antitrypsin [10]. IPSCs can also develop into liver organoids under proper conditions [38]. Following the induction of hepatic endoderm from human iPSCs, the hepatic endoderm together with human umbilical vein endothelial cells and human mesenchymal stem cells were cultured in specific culture system and ultimately formed liver organoids [38]. After transplanted into the cranial window of non-obese diabetic/severe combined immunodeficient mouse, the liver buds efficiently connected to host vascular networks in vivo, which was essential to the liver bud maturation [38]. Interestingly, the transplants displayed function maturation, including the production of albumin and human serum α-1-antitrypsin as well as drug metabolism activity in ketoprofen and debrisoquine [38].

### Pancrea organoids

In several studies, pancreatic organoids have been successfully established [10, 11, 39, 51]. A subgroup of progenitor cells with high aldehyde dehydrogenase (ALDH) activity ALDH^high^ formed organoids more efficiently with high expression of pancreatic progenitor markers, such as PDX1, pancreas associated transcription factor 1a, carboxypeptidase A1 and MYC in comparison with ALDH^low^ cells [11]. The differentiation to insulin-positive cells was observed after the organoids were transplantation into immunodeficient mice [11], and insulin was expressed accompanied with several functional endocrine markers of PDX1, islet amyloid polypeptide, NKX6.1 and synaptophysin [11]. In another study, pancreatic progenitor cells derived from hPSCs formed pancreatic organoids with the expression of exocrine markers (amylase and chymotrypsin C), ductal markers (SOX9 and cytokeratin 19 (CK19) and cystic fibrosis transmembrane conductance regulator), the epithelial marker E-cadherin and the progenitor marker NKX6.1 [39]. In terms of structures, these organoids displayed specific ductal morphology with microvilli and tight junction, and contained a hollow central tubular structure with a basal lamina and apical-basal polarity [39]. At functional level, carbonic anhydrase activity and enzymatic activity of amylase, trypsin and elastase were detected in the pancreatic organoids [39].

### Prostate organoids

The establishment of prostate organoids has been described in several studies [16, 52, 53]. A previous research reported a well-defined protocol to develop prostate organoids for long term from luminal and basal prostatic epithelial lineages [16]. Chua CW and colleagues showed the generation of prostate organoids from single luminal progenitor cells in the absence of stroma [52]. HPSCs-derived prostate organoids displayed a network of epithelial-like ducts, which consisted of monolayer epithelium with a central lumen and encircled basement membrane and stromal cells [53]. These organoids exhibited prostatic-like structures with typical expression of prostate specific antigen and androgen receptor as well as the capacity of response to testosterone and production of secretory proteins [53].

### Brain organoids

Human brain was an intricate organ and many physiological and pathological processes remain elusive. Brain organoids have been developed and hold great potential to reveal the complex process of development and abnormality. Up to now, a variety of neural organoids [17, 54] have been generated including the whole brain [24, 55–59] and sub-brain regions [60], such as of hypothalamus [61], adenohypophysis [62], midbrain [63], cerebellum [40] and hippocampus [64].

Mouse ESCs-derived adenohypophysis organoids have been developed with similar structure and specific function [62]. ESCs with hedgehog signaling treatment were promoted to differentiate to hypothalamic neuroectoderm, and various endocrine cells were produced [62]. In response to corticotrophin releasing hormone, the corticotrophs secreted adrenocorticotropic hormone in reaction [62].

Midbrain organoids from PSCs have been reported by Jo J’s group [63]. These organoids contained different layers of neuronal cells with the expression of midbrain-specific markers of homeobox transcription factor 1 alpha, CORIN, forkhead box A2 and orthodenticle homeobox 2 (OTX2), and it was observed that there were functionally mature midbrain dopaminergic neurons and dopamine production in the midbrain-like 3D structures [63]. In addition, these organoids produced neuromelanin-like granules that structurally mimicked those in human nigra tissues [63].

Human ESC-derived cerebellum organoid model was successfully built through chemically-defined 3D culture system [40]. Following dissociation and reaggregation, robust differentiation of the aggregates to nerve was observed with the expression of midbrain-hindbrain markers after 2 weeks [40]. FGF2 exposure inhibited the forebrain markers OTX2 and sine oculishomeobox homolog 3, but upregulated FGF8 and WNT1 [40]. Until 21 days, the hESC aggregates were transferred from 96-well plate to petri dish [40]. Subsequently, hESCs with FGF2 treatment expressed kirre like nephrin family adhesion molecule 2, which was one of the earliest markers for cerebellar plate neuroepithelium [40].

Mellios’s group employed human cerebral organoids to reveal deficits in neuronal migration and neurogenesis in neural progenitors with the deficient of methyl-CpG binding protein 2 [65]. It has been recently reported that vascularized brain organoids were successfully generated from a patient’s own iPSCs by Pham MT’s group [66].

## Application of organoid technology

### Organoids in the study of tissue development

Organoids hold great promise for basic biomedical research. This novel tool has now enabled researchers to study in vitro organogenesis, lineage specification and tissue homeostasis. Induced human intestinal organoids (iHIOs) can be generated to mimic the human intestine through exposure to a series of growth factors to mimic embryonic intestinal development [67]. Activin-A induces definitive endoderm formation, FGF/WNT induces posterior endoderm patterning, hindgut specification, and morphogenesis. FGF/WNT also promotes a pro-intestinal culture system to encourage intestinal growth, morphogenesis, and cytodifferentiation. After 28 days in culture, iHIOs were found to express intestinal stem cell markers and consist of polarized, columnar epithelium patterned into a villus-like structure that collectively exhibited similar morphology to human intestinal epithelium [68].

McCracken et al. reported that the development of human gastric organoids extremely resembled stomach organogenesis in vivo, experiencing a series of critical stages including definitive endoderm induction, posterior foregut specification and anterior gut tube formation, specification and patterning into fundus or antrum, and epithelial growth and differentiation [36]. One month-old organoids remarkably resembled human fetal stomach tissues in transcriptional profile, and contained surface mucous cells with mucin 5AC expression, mucin 6^+^ antral gland cells, SOX9^+^ proliferative progenitor zone, Lgr5^+^ cells and endocrine cells [36]. It was observed that high levels of epidermal growth factor (EGF) inhibited the expression of neurogenin 3 (NEUROG3) (a proendocrine transcription factor) and suppressed endocrine cell formation, while low level of EGF or *NEUROG3* overexpression promoted the differentiation of endocrine cells in gastric organoids [36]. LBOs derived from hPSCs, containing mesoderm and pulmonary endoderm, formed branching airway and early alveolar architectures in 3D culture system [7]. According to expression and structural analyses, the branching structures in LBOs are tantamount to fetal lung at the mid-term of human gestation [7]. Human neural organoids from embryonic stem cells were established in the rotary cell culture system to explore whether microgravity affected the generation and maintenance of these organoids [69]. The results showed that neural organoids could be successfully generated and maintained in the condition of microgracity, but there were still some changes in the level of cortical markers and rostral-caudal neural patterning genes relative to the organoids generated in standard conditions [69].

### Organoids in disease modelling

#### Infectious disease

Respiratory tract infection in infants is mainly caused by respiratory syncytial virus, tropism of which includes human alveolar epithelial cells and ciliated cells [70]. Human airway epithelial cells infected with respiratory syncytial virus swell and detach from the epithelium, and the detached cells contribute to obstruction of small airways which is a common manifestation of this infectious disease [70, 71]. It has been demonstrated that infecting hPSC-derived LBOs with respiratory syncytial virus contributed to swelling and detachment as well as shedding of infected cells into organoid lumens, which resembled the phenomenon observed in human lungs [7].

The popular organoid technology facilitates the investigation of the interaction between the gastrointestinal and enteric viruses [72]. It has been demonstrated that gastric organoids can be employed to model the pathophysiologic response of stomach to *Helicobacter pylori* infection [36]. Microinjection of *Helicobacter pylori* into the epithelial lumen of gastric organoid induced epithelial response, including remarkable phosphorylation of c-Met and robustly enhanced proliferation [36]. In addition, *Helicobacter pylori* virulence factor cytotoxin associated gene A (CagA) translocated into epithelial cells and combined with c-Met receptor [36]. When infected with a strain of *Helicobacter pylori* without CagA, organoids were nonreactive to the infection with no epithelial response [36]. These phenomena revealed that CagA was critical for *Helicobacter pylori*-mediated pathophysiological responses in gastric organoid [36].

Recently, Baktash and his colleges employed polarized hepatoma organoids to mimic the entry of hepatitis C virus (HCV) to hepatocytes, revealing that HCV engage and enter host cells through an active and multi-step procedure [73]. HCV firstly localized with the entry factors scavenger receptor B1, cluster of differentiation 81 and epidermal growth factor receptor (EGFR), and subsequently actin-dependently gathered at the tight junction [73]. Furthermore, HCV associated with another two factors claudin-1 and occludin at the tight junction and enter into hepatocytes through clathrin-mediated endocytosis in a dynamic process requiring EGFR [73].

Zika virus (ZIKV) infection can contribute to some severe syndromes, including Guillain–Barré syndrome and congenital Zika syndrome [74]. ZIKV can infect and destroy cells from central nervous system, including neurons, progenitors and glial cells [74]. Neural progenitor cells are particularly vulnerable to ZIKV infection, which results in neurogenesis impairment, cell death and ultimately microcephaly after birth [74]. ZIKV-infected cerebral organoids have been propagated by Novitch BG and colleagues [59]. After ZIKV infection, widespread progenitor apoptosis and whole growth restriction were observed, and inflammatory responses were switched on in direct and indirect manners in organoids, resulting in neurodegeneration [59].

#### Genetic disease

Organoids can serve as an extraordinary platform for the study on the biology of some genetic diseases. For instance, retinitis pigmentosa is an inherited and irreversible disease, and majorly caused by mutations in *PRGR* gene [75]. Patient-derived retinitis pigmentosa organoids harboring *PRGR* mutations have been developed from iPSCs, and faithfully recapitulated the defects of morphology, localization, transcriptional profiling and electrophysiological activity in photoreceptor as well as shorted cilium [75].

Considering that *disrupted in schizophrenia 1* (*DISC1*) interruption had been implicated in mental illness, Srikanth and his colleges employed the 3D model to investigate the role of *DISC1* in psychiatric disease [76]. They developed cerebral organoids from *DISCI*-disrupted and isogenic wild-type human pluripotent stem cells, respectively [76]. *DISC1*-mutant organoids exhibited disorganized structural morphology and weakened proliferation with changes of cell fate markers, interneuron markers and regulators of neuronal migration [76].

*Hermansky*–*Pudlak syndrome 1* (*HPS1*) mutation partly accounts for the formation of intractable pulmonary fibrosis [7]. Introduction of *HPS1* mutation to LBOs formed lung organoids with lung fibrosis, in which extracellular matrix and mesenchymal cells accumulated [7]. This observation signifies that organoids can serve as a potential model to recapitulate fibrotic lung disease.

#### Cancer

Organoids have tremendous promise for modelling human cancers and exhibit their utility for translational and clinical cancer research [77, 78]. Up to now, organoids have been successfully developed from multiple cancer types, including gastric cancer [19, 20], intestinal cancer [19], liver cancer [21], pancreatic cancer [79–81], prostate cancer [82–84], bladder cancer [23] and breast cancer [22, 85].

Gastric cancer organoids faithfully recapitulate important characteristics of the corresponding parent tumors as exemplified by architectures, the expression of typical gastric cancer markers and the presence of various prevalent mutations in gastric cancer [20]. In addition, colorectal cancer organoids showed remarkable resemblance with the primary tumors in the aspects of morphology, mutational landscape and transcriptomic profiling [19]. Broutier and colleagues demonstrated that primary liver cancer organoids retained typical architectures and similar transcriptomic profiles to parent tumors [21]. Seino et al. detected pancreatic carcinoma-related gene alterations in corresponding tumoroids, and observed tumor formation that resembled the derived cancer in organoid-transplants [79].

Gao’s group demonstrated that there were a diversity of characteristic copy number alterations and mutations detected in both organoid lines and corresponding parent prostate cancer as well as similar histological patterns in organoid xenoplants [82]. When transplanted into mice, these organoid lines displayed histological patterns found in parent tumors [82]. Histological analyses on bladder cancer organoids reported not only the remarkable resemblance in mutational profiles between these organoids and the corresponding derived tumors, but also cancer evolution with some genomic changes in vitro [23]. Besides, breast cancer organoid lines also resembled the parent tumors in morphology, histopathology and gene profiles [22].

### Organoids in drug development and precision medicine

As near-physiological architectures, organoids faithfully recapitulate the primary tumors and can faithfully recapitulate drug responses [19, 20, 33, 86, 87] as well as even help optimize therapy strategies for each patient individual. Healthy organoids can be exploited to assess the drug toxicity, such as hepatotoxicity experimental compounds [32, 88, 89], cardiotoxicity [87, 90] and nephrotoxicity [91].

#### Infectious diseases

Drug testing has been conducted in ZIKV-infected cortical organoids [59]. 25-hydroxycholesterol (25HC), which obstructs viral entry through suppressing membrane fusion, was assessed in the infectious organoid models [59]. After the addition of 25HC to organoid cultures, both ZIKV mRNA and whole number of ZIKV E positive cells dramatically decreased among organoids, with no amelioration on cell death [59]. Considering that inhibition or competitive combination of the AXL receptor on neural progenitors can restrict ZIKV entry or spread, the molecular inhibitor of AXL (R428) or the antibodies to AXL have also been tested in organoids, showing that R428 had mild negative effects on ZIKV mRNA level but antibody treatment had no significant effects on ZIKV mRNA level [59]. Administration of duramycin or ivermectin remarkably decreased ZIKV mRNA and ZIKV envelope protein positive cells, but azithromycin can not reduce the level of ZIKV expression [59]. The results indicated that cortical organoids can be employed to distinguish drugs for ZIKV infection in developing human nervous system and identified promising compounds.

#### Genetic diseases

Cystic fibrosis (CF) organoids have been successfully established to explore potential therapy strategies for patients [26, 39, 92]. Compound screen using organoid as a platform, indicated that two types of small-molecule compounds, including cystic fibrosis transmembrane conductance regulator (CFTR) correctors for improving cellular processing and CFTR potentiators for gating function of the CFTR protein, effectively significantly rescued CF phenotype [39]. Furthermore, it was also demonstrated that the modification of chemically modified mRNA supplemented CFTR gene and restored CFTR function in organoids [39]. Therefore, the newly spring-up culture system represents a novel approach to drug screening for genetic diseases.

#### Cancer

Recently, metastatic gastrointestinal cancer organoids have been exploited to assess the potential of the novel technology in prediction of treatment response among patients [19]. The results demonstrated that these organoids could faithfully recapitulate treatment responses of gastrointestinal cancers with high sensitivity (100%) and specificity (93%) [19]. In addition, Therese Seidlitz’s group identified that human gastric cancer organoids recapitulated the distinct responses to conventional chemotherapeutics [20]. According to another study, the combination of navitoclax (a preclinical B-cell CLL/lymphoma 2 (BCL2)/BCLXL inhibitor), afatinib and selumetinib potently induced cell death in comparison with monotherapy of these drugs, signifying a possible alternative treatment strategy for colorectal cancer patients [93]. In accordance with clinical observation, androgen receptor (AR)-amplified prostate cancer organoids responded efficiently to the AR inhibitor enzalutamide, while AR-negative prostate cancer organoids were irresponsive to this drug [82]. Apart from testing the efficacy of a known therapy, organoid lines can also be employed to test the effectiveness of treatment on unknown mutations. For instance, imatinib treatment was effective for an unknown mutation in exon 3 of the c-KIT receptor [20].

Organoid technology exerts enormous potential in precision medicine. According to Rubin’s study, the combination therapy of vorinostat and buparlisib is the most effective for uterine carcinosarcoma patients harboring driver mutations in *phosphatidylinositol*-*4,5*-*bisphosphate 3*-*kinase catalytic subunit alpha* (*PIK3CA*) and *phosphatase and tensin homolog* (*PTEN*) among all combination strategies, and the treatment of buparlisib plus olaparib displayed strongest anti-tumor effects in the endometrial adenocarcinoma organoids organoid with mutations in *PIK3CA* and *PTEN* [94]. For late-stage colorectal cancer with *KRAS* and *TP53* mutations, the treatment of trametinib and celecoxib was the most effective combinational strategy [94]. In addition, the combination therapy of hedgehog signal inhibitors plus 5-fluorouracil (5-FU) or irinotecan exerted strong anti-tumor effects for 5-FU or irinotecan-resistant colorectal cancer organoids [95], signifying that hedgehog signal inhibitors were effective combinational candidates for colorectal cancer patients with resistance to 5-FU or irinotecan.

### Regeneration medicine

Modern regeneration medicine aims to replace the diseased tissues with corresponding healthy tissues through allogenic transplantation. However, the deficiency in the supply of healthy donor tissues and the inherent immunological rejection pose challenges to the survival and function of transplanted tissues in recipient patient body for long term. Organoid technology endows researchers with the ability of developing isogenic or human lymphocyte antigen-matched organoids from minuscule amounts of patient biopsies or readily accessible tissues.

Many studies have demonstrated the potential of organoid technology to provide an alternative approach to organ replacement strategies for several tumor types. Following transplantation to mice harboring colonic injury, colonic epithelium was observed in mature intestinal organoids with the formation of epithelial crypt-like architectures and the expression of region-specific differentiation markers, leading to colon repair after injury [96]. When transplanted into mice experiencing acute liver failure, liver organoids generated from umbilical cord-derived endothelial and mesenchymal cells as well as the endothelial cell-derived human iPSC dramatically rescued the hepatic functions and enhanced the survival of diseased mice [34]. Human cholangiocytes, which were isolated and propagated from the biliary tree, reconstructed extrahepatic cholangiocyte organoids which were quite similar to the parent counterparts in ultrastructure, transcriptomic profile and functional properties [97]. After transplantation under the kidney capsule of mice, organoids formed tubular architecture with the expression of the key biliary marker CK19 [97]. Vascularization is an essential event for maturation and function of organoids in vivo. It has been reported that host-derived vascularization formed in iPSC-derived kidney organoids in fully defined conditions without any exogenous vascular endothelial growth factor. Progressive morphogenesis, including functional glomerular perfusion in function as well as connection to pre-existing vascular system, glomerular basement membrane and fenestrated endothelial cells in structure, was observed in these organoids after transplanted under the kidney capsule [35].

Additionally, organoid technology also represents an alternative approach for degeneration diseases, as exemplified by retinal degeneration and blindness, which was enlightened by encouraging stem cell-based treatment clinical trials that focused on the replacement therapy of degenerated retinal cell types with stem cells [98]. Finally, patient organoids harboring genetic defects can be repaired by modern genome-editing techniques in vitro to generate healthy organoids and subsequently transplanted back into patients for transplantation use. Dekkers’s group utilized clustered regularly interspaced short palindromic repeats (CRISPR)/CRISPR-associated protein 9 (Cas9) gene editing to correct *CFTR* mutations in intestinal organoids from CF patients to generate healthy organoids [26]. Another example is retinitis pigmentosa organoid, in which correction of retinitis pigmentosa guanosine triphosphatase regulator mutation through CRISPR-Cas9 technique normalized gene expression, rectified photoreceptor structure, and reversed electrophysiological property [75].

## Current limitations

Current organoid technology still represents an imperfect version. Firstly, organoids only contain epithelial layer without tissue microenvironment, such as immune system and nervous system [86]. Secondly, fully maturation to adult organs or tissues is a bottleneck required to be addressed. Thirdly, another limitation is the dependence on the extracellular matrix Matrigel or basement membrane extract of current organoids, which is produced from mouse tumor lines and thus might be unsuitable for human. Matrigel could also hamper drug penetration and be adverse to the potential of organoids in drug screens. Fourthly, culture medium needs to be further refined for long-term expansion of some organoids. Fifthly, growth factors or molecular inhibitors in culture medium might have some effects on drug responses of organoids. Further efforts will be urgently exerted to solve these problems.

## Conclusion

In spite of current limitations, this promising organoid technology holds great potential in accurately modelling organ development and human diseases, and it can serves as an extraordinary platform for therapy response-prediction, drug development and personalized medicine. Furthermore, organoid technology represents an excellent alternative for transplantation use. Future efforts will doubtless improve this novel tool for clinical application.

## Abbreviations

ALDH: aldehyde dehydrogenase
AR: androgen receptor
AT1: alveolar type 1
BCL2: B cell CLL/lymphoma 2
CagA: cytotoxin associated gene A
Cas9: CRISPR-associated protein 9
CF: cystic fibrosis
CFTR: cystic fibrosis transmembrane conductance regulator
CK19: cytokeratin 19
CRISPR: clustered regularly interspaced short palindromic repeats
DISC1: disrupted in schizophrenia 1
DMEM: Dulbecco’s modified Eagle’s medium
EGF: epidermal growth factor
EGFR: epidermal growth factor receptor
ESCs: embryonic stem cells
FGF4: fibroblast growth factor 4
HCV: hepatitis C virus
HPS1: Hermansky–Pudlak syndrome 1
hPSCs: human pluripotent stem cells
iHIOs: induced human intestinal organoids
iPSCs: induced pluripotent stem cells
LBO: lung bud organoids
NEUROG3: neurogenin 3
OTX2: orthodenticle homeobox 2
PDX1: duodenal homeobox 1
PIK3CA: phosphatidylinositol-4,5-bisphosphate 3-kinase catalytic subunit alpha
PTEN: phosphatase and tensin homolog
SOX2: sex determining region Y-box 2
ZIKV: zika virus
25HC: 25-hydroxycholesterol
3D: three-dimensional
5-FU: 5-fluorouracil
